# Characteristics of Hepatitis B Virus, Hepatitis C Virus, and Syphilis Coinfection in People With HIV/AIDS Contracted Through Different Sources: Retrospective Study

**DOI:** 10.2196/46750

**Published:** 2024-02-27

**Authors:** Rongrong Yang, Rui Yuan, Xien Gui, Hengning Ke, Ke Zhuang, Hui Hu, Ling Li, Ling Feng, Xingxia Yu, Yajun Yan, Mingqi Luo

**Affiliations:** 1 Zhongnan Hospital of Wuhan University Wuhan China; 2 Center for AIDS Research Wuhan University Wuhan China; 3 Animal Biosafety Level-III Laboratory at the Center for Animal Experiment State Key Laboratory of Virology Wuhan University Wuhan China; 4 Department of Emergency, Renmin Hospital, Wuhan University Wuhan China

**Keywords:** acquired immunodeficiency syndrome, AIDS, human immunodeficiency virus, HIV, hepatitis B virus, HBV, hepatitis C virus, HCV, syphilis

## Abstract

**Background:**

The burden of hepatitis B virus (HBV), hepatitis C virus (HCV), and syphilis coinfections remains disproportionately high among people living with HIV/AIDS. Hubei province is located in central China, where there are distinct regional characteristics of the distribution of people living with HIV/AIDS acquired via diverse transmission routes and the AIDS epidemic itself.

**Objective:**

We aimed to estimate the magnitude of HBV, HCV, or syphilis coinfections among people living with HIV/AIDS with blood-borne transmission, which includes former paid blood donors, contaminated blood recipients, and intravenous drug users, as well as among people with sex-borne HIV transmission (including heterosexual people and men who have sex with men) and people with mother-to-child HIV transmission.

**Methods:**

From January 2010 to December 2020, people living with HIV/AIDS were tested for hepatitis B surface antigen (HBsAg), HCV antibodies, and syphilis-specific antibodies. The positive patients were further tested for HBV markers, HBV DNA, and HCV RNA, and received a rapid plasma reagin circle card test. All people living with HIV/AIDS were first divided into transmission groups (blood, sex, and mother-to-child); then, people with blood-borne HIV transmission were divided into former paid blood donors, contaminated blood recipients, and intravenous drug users, while people with sex-borne HIV transmission were divided into heterosexual people and men who have sex with men.

**Results:**

Among 6623 people living with HIV/AIDS, rates of chronic HCV infection were 80.3% (590/735) in former paid blood donors, 73.3% (247/337) in intravenous drug users, 57.1% (444/777) in contaminated blood recipients, 19.4% (21/108) in people with mother-to-child HIV transmission, 8.1% (240/2975) in heterosexual people, and 1.2% (21/1691) in men who have sex with men. Chronic HBV infection rates were similar among all people with blood-borne HIV transmission. However, compared to heterosexual people, the chronic HBV infection rate was greater in men who have sex with men (213/1691, 12.6% vs 308/2975, 10.4%; χ^2^_1_=5.469; *P*=.02), although HBV exposure was less common (827/1691, 48.9% vs 1662/2975, 55.9%; χ^2^_1_=20.982; *P*<.001). Interestingly, the combination of HBsAg and hepatitis B e antigen (HBeAg) was found in 11 patients with sex-borne HIV transmission, but in 0 people with blood-borne HIV transmission (11/196, 5.6% vs 0/521, 0%; χ^2^_1_=29.695, *P*<.001). In people with sex-borne HIV transmission, the proportions of patients with a syphilis titer ≥1:16 and neurosyphilis were 8.6% (105/1227) and 7.8% (37/473), respectively, whereas these values were 0 in people with blood-borne HIV transmission.

**Conclusions:**

In people living with HIV/AIDS, HCV transmission intensity was significantly associated with specific exposure modes of blood or sexual contact. The rate of chronic HBV infection among men who have sex with men was higher than in any other population. Attention should be paid to the high prevalence of neurosyphilis in people living with HIV/AIDS who contract HIV by sexual intercourse.

## Introduction

According to a 2020 World Health Organization (WHO) report, 37.6 million people are infected with HIV worldwide [1]. Hepatitis B virus (HBV), hepatitis C virus (HCV), and syphilis infection are also all major public health problems worldwide. HBV, HCV, and syphilis are the 3 most common infections among people living with HIV/AIDS all over the world and share similar transmission routes [2,3]. The burden of HBV, HCV, and syphilis infection in people living with HIV/AIDS is even larger than in the general population. The ever-increasing burden of these infections has become a growing concern [4]. The prevalence of HBV, HCV, and syphilis coinfections in people living with HIV/AIDS is 8% to 12.2% [5-7], 33% to 80% [8,9], and 40.5% [10], respectively.

With the apparent efficiency of infectious illness prevention and control, as well as the recent introduction of direct-acting antiviral medicines, the WHO has proposed long-term worldwide prevention and control as the ultimate goal. A full assessment of HBV, HCV, and syphilis coinfection in people living with HIV/AIDS will aid in the development of a phased management plan that will contribute to the global eradication of hepatitis B disease by 2030 [11] and make HCV eradication a reality [12].

As the distribution of high-risk groups varies by country, so does the burden of HBV, HCV, and syphilis coinfection. Currently, the characteristics of the AIDS epidemic and the distribution of high-risk populations have changed. On the one hand, people who became infected with HIV as a result of nonroutine HIV screening of blood products still have HBV or HCV-related diseases; on the other hand, with the increasing transmission of AIDS among men who have sex with men, the pressure to control syphilis transmission differs from the blood transmission era of AIDS and heterosexual unprotected contact. Although HIV, HBV, HCV, and syphilis infections have been reported in a wide range of populations, including prisoners [13], blood donors [14], parturient women [15], and even inpatients [16], data on comparative infection rates among different populations in the same region remain scarce. A more extensive comparison of the prevalence and transmission intensity of coinfection with HBV, HCV, and syphilis among different risk groups will serve as the foundation for a comprehensive assessment of the disease burden in the HIV-positive community.

The Hubei provincial region includes populations of all high-risk HIV-infected groups and can be described as an appropriate place to examine the coinfection status of HBV, HCV, and syphilis in different populations of people living with HIV/AIDS in the same province. Hubei is located in central China. Poor rural farmers supplied plasma under unsanitary conditions in the 1990s, resulting in HIV infection among former paid blood donors and contaminated blood recipients [17-19]. Later, as a result of imported cases, an AIDS epidemic began to spread among intravenous drug users, heterosexual people, and men who have sex with men. As a result, the Hubei province AIDS epidemic has distinct geographical characteristics.

In this study, HIV-infected people were classified according to 3 classical sources of transmission: blood, sex, and mother-to-child transmission (MTCT); further subgroup analysis was performed in the blood-source and sex-source populations, with the goal of comparing differences in the disease prevention and control burden of HIV combined with HBV, HCV, and syphilis among various high-risk groups. This can not only maximize the allocation of comprehensive disease prevention and control expenditures in resource-rich locations, but it can also serve as a reference point for resource-limited places to achieve larger returns with a small investment, allowing for progress in reaching the final goal.

## Methods

### Study Population

From January 2010 to December 2020, a total of 6623 people living with HIV/AIDS were tested for hepatitis B surface antigen (HBsAg), HCV antibodies, and syphilis-specific antibodies at Zhongnan Hospital of Wuhan University. Patients who tested positive for HBsAg were also tested for HBV markers and HBV DNA. HCV RNA was investigated further in HCV antibody–positive patients, and the rapid plasma reagin circle card test was used in syphilis antibody–positive patients. First, all people living with HIV/AIDS were divided into 3 groups based on their HIV acquisition route: blood, intercourse, or MTCT. They were then redivided into former paid blood donors, contaminated blood recipients, and intravenous drug users in the blood-borne transmission group, and heterosexual people and men who have sex with men in the sex-borne transmission group. The inclusion criteria were (1) confirmed HIV infection and (2) a definite route of HIV acquisition. The exclusion criteria were (1) having 2 or more high-risk behaviors for HIV infection, (2) acute HBV infection, and (3) acute HCV infection.

### Ethical Considerations

This study was a secondary analysis of a preexisting data set, and ethical review and approval were waived for this study by the institutional review board of the work unit (Zhongnan Hospital of Wuhan University) of the first author (RY). Informed consent was obtained, and all the analyzed data were anonymous.

### Study Protocol

Differences in HBV, HCV, and syphilis transmission intensity were analyzed in groups divided according to the 3 classical transmission routes of HIV acquisition: blood, sex, and MTCT. Then, in the blood-origin population, the participants were further divided into former paid blood donors, contaminated blood recipients, and intravenous drug users, whereas the sex-origin population was further divided into heterosexual people and men who have sex with men. Differences in HBV or HCV coinfection rates and characteristics of syphilis infection were compared in the different high-risk groups with the same HIV origin.

### Laboratory Testing

Two rapid assays or joint detection modes were used to assess HIV status: enzyme immunoassay (EIA) and Western blotting. Anti-HCV, HBsAg, and *Treponema pallidum* antibody detection testing were done with a third-generation EIA (Shanghai Kehua Bio-Engineering). HBV markers, including HBsAg, hepatitis B surface antibody (anti-HBs), hepatitis B e antigen (HBeAg), hepatitis B e antibody (anti-HBe), and hepatitis B core antibody (anti-HBc), were examined with an enzyme-linked immunosorbent assay (ELISA). HCV RNA was tested using a polymerase chain reaction fluorescence detection kit (Shanghai Kehua Bio-Engineering) with a lower limit of detection of 20 copies/ml. The conventional rapid plasma reagin (RPR) circle card test, which uses cardiolipin antigen with a carbon particle to detect reagin, was used to determine the titer of syphilis. The experimental procedure and the analysis of results were performed according to the manufacturer’s instructions.

### Definitions

Passive immunization is defined as the presence of anti-HBe and a vaccination history. Natural immunization is characterized as having a vaccine history and HBV markers in addition to anti-HBe, or not having a vaccination history but having HBV markers. Natural HBV exposure was defined as not having had a hepatitis B vaccine but having one or more HBV indicators or having received a hepatitis B vaccine and having one or more HBV markers except for anti-HBe.

As in a previous report [20], a reactive cerebrospinal fluid (CSF) venereal disease research laboratory test result, a CSF white blood cell count of >20 cells/l, or both were used to define neurosyphilis. Patients who had received penicillin previous to lumbar puncture or who had missing data, such as from a full skin examination or a complete neurological examination, were excluded from this study.

### Statistical Analysis

Differences in characteristics across groups were compared using the Wilcoxon rank-sum test (2-group comparisons) and the Kruskal-Wallis rank test (≥3 group comparisons). The Pearson chi-square test was used to compare categorical variables across groups. A 2-tailed *t* test was applied to compare differences in measurement data. Statistical calculations were performed using SPSS (version 17.0; IBM Corp), with a *P* value <.05 considered significant.

## Results

### Demographic Characteristics of People Living With HIV/AIDS

This study investigated 6623 people living with HIV/AIDS from January 2010 to December 2020, of whom 71.27% (4720/6623) were male. Their average age was 37.6 (SD 12.3) years, and most (n=5838, 88.15%) were aged 18 to 55 years. Most (n=5805, 87.65%) of the individuals were receiving antiretroviral therapy (ART), and the average CD4+ T lymphocyte count was 226 (SD 86) cells/ul. The routes of HIV acquisition for all individuals are shown in Table 1. On the whole, the positive rates for HBsAg, anti-HCV, and antisyphilis antibodies were 10.92% (723/6623), 23.6% (1563/6623), and 21% (1391/6623), respectively.

**Table 1:** Demographic characteristics of the study population (N=6623).

Characteristics			People living with HIV/AIDS
**Sex**			
	Male, n (%)	4720 (71.27)	
	Female, n (%)	1903 (28.73)	
**Age group (years), n (%)**			
	≤18	138 (2.08)	
	19-35	2974 (44.9)	
	36-55	2864 (43.24)	
	≥56	647 (9.77)	
**HIV transmission route, n (%)**			
	Former paid plasma donation	735 (11.1)	
	Contaminated blood transfusion	777 (11.73)	
	Intravenous drug use	337 (5.09)	
	Mother-to-child transmission	108 (1.63)	
	Men who have sex with men contact transmission	1691 (25.53)	
	Heterosexual contact transmission	2975 (44.92)	
CD4+ T lymphocyte count (cells/ul), mean (SD)			226 (86)
Antiretroviral therapy coverage, n (%)			5805 (87.65)
Hepatitis B surface antigen positive, n (%)			723 (10.92)
Anti–hepatitis C virus positive, n (%)			1563 (23.6)
Hepatitis C virus RNA detectable, n (%)			1112 (16.79)
Antisyphilis antibody positive, n (%)			1391 (21)
Positive in rapid plasma reagin circle card test, n (%)			520 (7.85)

**Figure 1:**
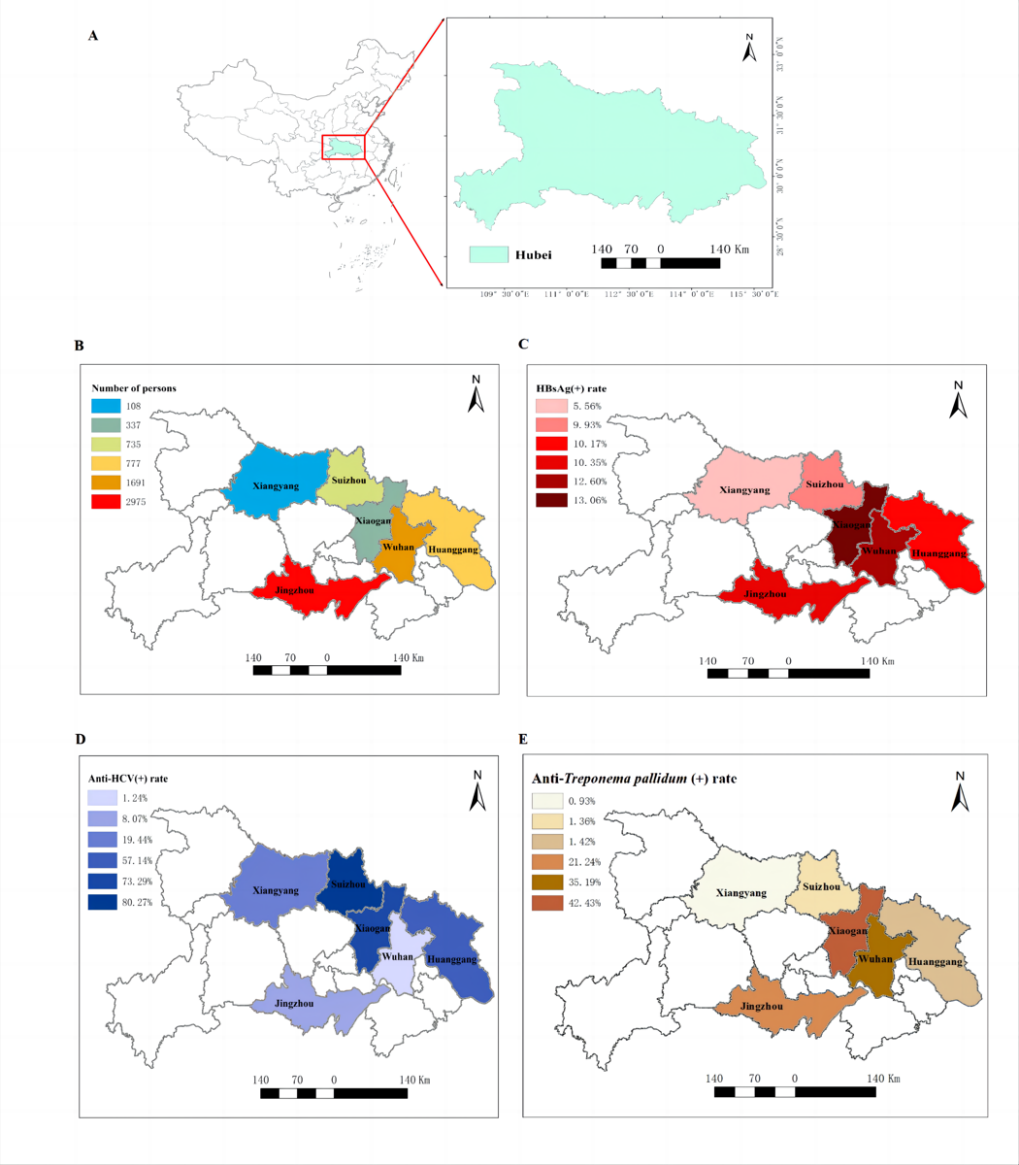
Maps of the study area, sample sites, and prevalence of hepatitis B virus, hepatitis C virus (HCV), or syphilis coinfections among people living with HIV/AIDS. Figures 1A and 1B depict the study location and population geographic distribution. Figures 1C, 1D, and 1E depict the overall positive rates of hepatitis B surface antigen (HBsAg), HCV, and syphilis, respectively.

Figure 1A and Figure 1B depict the study location and population geographic distribution, whereas Figure 1C, Figure 1D, and Figure 1E depict the overall positive rates for HBsAg, HCV, and syphilis, respectively.

### Coinfection Rates of HBV and HCV in Patients Who Acquired HIV via the 3 Routes Of Transmission

According to the route of HIV acquisition, the 6623 patients were divided into those with blood-borne transmission (n=1849), MTCT (n=108), and sex-borne transmission (n=4666). The coinfection rates of HBV were 10.6% (196/1849), 5.56% (6/108), and 11.17% (521/4666), respectively. There was no significant difference in HBV coinfection rate among the 3 groups (χ^2^_2_=3.680; *P*=.16). However, the coinfection rates of HCV in people with blood-borne transmission, MTCT, and sex-borne transmission were 69.28% (1281/1849), 19.44% (21/108), and 5.59% (261/4666), respectively. These HCV infection rates were significantly different among the 3 groups (χ^2^_2_=2980.043; *P*<.001). People with blood-borne HIV transmission had a higher prevalence of HCV coinfection than people with MTCT (1281/1849, 69.28% vs 21/108, 19.44%; χ^2^_1_=113.812; *P*<.001) and people with sex-borne HIV transmission (1281/1849, 69.28% vs 261/4666, 5.59%; χ^2^_1_=2973.003; *P*<.001); people with MTCT had a higher prevalence of HCV coinfection than people with sex-borne transmission (21/108, 19.44% vs 261/4666, 5.59%; χ^2^_1_=36.434; *P*<.001).

### Coinfection Rates of HBV and HCV in Different Populations With Same Origin of HIV

According to differences in risk factors, 1849 people living with HIV/AIDS who acquired HIV by blood were further divided into former paid blood donors (n=735), contaminated blood recipients (n=777), and intravenous drug users (n=337). The coinfection rates of HBV were 9.9% (73/735), 10.2% (79/777), and 13.1% (44/337), respectively. There was no significant difference in the HBV coinfection rate among individuals who acquired HIV through blood transmission (χ^2^_2_=2.645; *P*=.27). However, HCV infection rates in former paid blood donors, intravenous drug users, and contaminated blood recipients were 80.3% (590/735), 73.3% (247/337) and 57.1% (444/777), respectively, indicating a statistically significant difference between the three groups (χ^2^_2_=98.060, *P*<.001). Former paid blood donors had a higher prevalence of HCV coinfection than intravenous drug users (590/735, 80.3% vs 247/337, 73.3%; χ^2^_1_=6.574; *P*=.01) and contaminated blood recipients (590/735, 80.3% vs 444/777, 57.14%; χ^2^_1_=93.462; *P*<.001), and intravenous drug users had a higher prevalence of HCV coinfection than contaminated blood recipients (247/337, 73.3% vs 444/777, 57.1%; χ^2^_1_=26.032; *P*<.001).

Similarly, 4666 people living with HIV/AIDS who acquired HIV by sexual contact were further divided into heterosexual people (n=2975) and men who have sex with men (n=1691). The coinfection rates of HBV in men who have sex with men were greater than in heterosexual people (213/1691, 12.6% vs 308/2975, 10.35%; χ^2^_1_=5.469; *P*=.02), but the reverse was the case for HCV coinfection (21/1691, 1.24% vs 240/2975, 8.07%; χ^2^_1_=95.113; *P*<.001).

### HBV Exposure and Chronic HBV Coinfection Among Individuals Who Acquired HIV Through Sexual Intercourse

The coverage of active immunization against HBV infection in men who have sex with men was greater than in heterosexual people (534/1691, 31.58% vs 574/2975, 19.29%; χ^2^_1_=89.860; *P*<.001), and the reverse was the case for passive immunity (614/1691, 36.31% vs 1354/2975, 45.51%; χ^2^_1_=37.440; *P*<.001). Compared with heterosexual people, the natural HBV exposure rate was lower in men who have sex with men (827/1691, 48.91% vs 1662/2975, 55.87%; χ^2^_1_=20.982; *P*<.001), but the chronic infection rate following natural exposure to HBV was greater in men who have sex with men (213/827, 25.76% vs 308/1662, 18.53%; χ^2^_1_=17.412; *P*<.001). These results are shown in Table 2.

**Table 2:** Hepatitis B virus (HBV) exposure and chronic HBV coinfection among individuals who acquired HIV through sexual intercourse.

			Heterosexual people (n=2975)		Men who have sex with men (n=1691), n (%)		Chi-square (*df*)	*P* value	
Passive immunization, n (%)			574 (19.29)		534 (31.58)		89.860 (1)	<.001	
**Natural immunization, n (%)**									
	Overall	1354 (45.51)		614 (36.31)		37.440 (1)		<.001	
	Anti-HBe^a^/anti-HBs^b^/anti-HBc^c^	254 (8.54)		125 (7.39)		1.897 (1)		.17	
	Anti-HBe/anti-HBc	515 (17.31)		363 (21.47)		12.189 (1)		<.001	
	Anti-HBs/anti-HBc	106 (3.56)		46 (2.72)		2.430 (1)		.12	
	Anti-HBc	479 (16.1)		80 (4.73)		132.176 (1)		<.001	
Natural HBV exposure, n (%)			1662 (55.87)		827 (48.91)		20.982 (1)	<.001	
Proportion of chronic HBV infection to HBV natural exposure population, n/N (%)			308/1662 (18.53)		213/827 (25.76)		17.412 (1)	<.001	
Chronic HBV infection, n (%)			308 (10.35)		213 (12.6)		5.469 (1)	.02	

^a^Anti-HBe: hepatitis B e antibody.

^b^Anti-HBs: hepatitis B surface antibody.

^c^Anti-HBc: hepatitis B core antibody.

### Uncommon Combination of HBV Markers Among Chronic HBV Infections

In people with blood- or sex-borne HIV transmission, 196 and 521 people, respectively, were found to be coinfected with HBV in this study. Three uncommon combinations of HBV markers were detected in these chronic HBV infection patients: HBsAg and anti-HBe positivity; HBeAg and anti-HBs positivity; and only HBsAg and HBeAg positivity. Among those with blood-borne HIV transmission, 6 of 196 (3.1%) people with HIV/HBV coinfection had HBsAg and anti-HBe positivity, compared to 12 of 521 (2.3%) people with sex-borne HIV transmission. The rates of HBsAg and anti-HBe positivity showed no statistical difference between the people with blood- or sex-borne HIV transmission (6/196, 3.1% vs 12/521, 2.3%; χ^2^_1_=0.334; *P*=.56). Similarly, 1 of 196 (0.5%) people with HIV/HBV coinfection among those with blood-borne HIV transmission had HBeAg and anti-HBs positivity, compared to 8 of 521 (1.5%) people with sex-borne HIV transmission. The rates of HBeAg and anti-HBs positivity showed no statistical difference between people with blood- or sex-borne HIV transmission (1/196, 0.5% vs 8/521, 1.5%; χ^2^_1_=1.208; *P*=.27). It was interesting that when testing HBV markers, coinfection with only HBsAg/HBeAg positivity was found in 11 patients with HIV/HBV among people with sex-borne HIV transmission, while none of the people with blood-borne HIV transmission (11/196, 5.6% vs 0/521, 0%; χ^2^_1_=29.695; *P*<.001) had this coinfection. The clinical indicators related to HBV infection in these 11 patients are shown in Table 3.

**Table 3:** Clinical indicators related to hepatitis B virus (HBV) infection among the 11 patients who were positive for only hepatitis B e antigen (HBeAg) and hepatitis B surface antigen (HBsAg).

Patient	Gender, age (years)	HBV infection history (years)	CD4^+^ T lymphocyte count (cells/ul)	ART^a^	ART regimen	ART duration	HBV DNA, IU/ml	Quantitative detection of HBsAg, IU/ml	Quantitative detection of HBeAg, s/co	ALT^b^ (U/L)	AST^c^ (U/L)
1	M, 33	10	23	N	N/A^d^	N/A	4.36×10^6^	>250	752.7	69	93
2	F, 26	5	280	Y	TDF^e^/3TC^f^/EFV^g^	5 years	<20	>250	438.45	12	24
3	M, 39	—^h^	220	N	N/A	N/A	<20	1.68	3.54	679	732
4	M, 39	—	36	N	N/A	N/A	3.36×10^7^	>250	1840.69	17	58
5	M, 51	—	57	N	N/A	N/A	2.33×10^4^	11.09	1.02	30	30
6	M, 32	3	2	N	N/A	N/A	6.57×10^5^	353.1	1109.894	16	26
7	M, 27	—	38	N	N/A	N/A	6.08×10^2^	10.04	1.46	46	44
8	M, 34	12	319	Y	AZT^i^/3TC/Lpv/r^j^	2 years	7.74×10^5^	11121	618.552	19	19
9	M, 28	—	14	N	N/A	N/A	6.04×10^8^	>250	1678	17	38
10	M, 31	—	9	Y	TDF/3TC/EFV	1 month	3.07×10^5^	40751	239.68	23	49
11	F, 62	14	220	Y	TDF/3TC/Lpv/r	2 years	<20	1837	1558.827	22	22

^a^ART: antiretroviral therapy.

^b^ALT: alanine aminotransferase.

^c^AST: aspartate aminotransferase.

^d^N/A: not applicable.

^e^TDF: tenofovir disoproxil fumarate.

^f^3TC: lamivudine.

^g^EFV: efavirenz.

^h^Not available (These patients did not receive HBsAg testing before but were diagnosed with chronic HBV during the implementation period of this study).

^i^AZT: zidovudine.

^j^Lpv/r: lopinavir/ritonavir.

### Characteristics of Syphilis Infection Among HIV Blood-Borne And Sex-Borne Transmission Population

The positive rate of *T pallidum* antibodies in the sex-borne transmission group was greater than in the blood-borne transmission group (1227/4666, 26.3% vs 164/1849, 8.87%; χ^2^_1_=239.499; *P*<.001). Among those individuals who were positive for *T pallidum* antibodies, the proportion of patients who were negative for RPR in the sex-borne transmission group was lower than in the blood-borne transmission group (754/1227, 61.45% vs 117/164, 71.34%; χ^2^_1_=6.046; *P*=.01). Similarly, among those who tested positive for RPR, the proportion of patients without symptoms was lower in the people with sex-borne transmission than in the people with blood-borne transmission (346/473, 73.15% vs 42/47, 89.36%; χ^2^_1_=5.932; *P*=.02). It was intriguing to note that high-titer syphilis infection and neurosyphilis only occurred in the people with sex-borne transmission. In people with sex-borne transmission, the proportion of patients with a syphilis titer ≥1:16 and neurosyphilis were 8.56% (105/1227) and 7.82% (37/473), respectively, indicating a statistically significant difference between the sex- and blood-borne transmission groups (105/1227, 8.56% vs 0/164, 0%; χ^2^_1_=15.180; *P*<.001 and 37/473, 7.82% vs 0/47, 0%; χ^2^_1_=3.958; *P*=.047). These results are shown in Table 4.

**Table 4:** The characteristics of syphilis infection among HIV blood-borne and sex-borne transmission populations.

			Blood-borne transmission population (n=1849), n (%)		Sex-borne transmission population (n=4666), n (%)		Chi-square (*df*)		*P* value
Syphilis antibodies			164 (8.87)		1227 (26.3)		239.499 (1)		<.001
**Rapid plasma reagin circle card test**									
	Negative	117 (71.34)		754 (61.45)		6.046 (1)		.01	
	Titer ≤1:8	47 (28.66)		368 (29.99)		0.123 (1)		.07	
	Titer ≥1:16	0 (0)		105 (8.56)		15.180 (1)		<.001	
**Symptoms**									
	None	42 (89.36)		346 (73.15)		5.932 (1)		.02	
	Fever	2 (4.26)		35 (7.4)		0.640 (1)		.04	
	Rash	2 (4.26)		40 (8.46)		1.016 (1)		.03	
	Lymphadenopathy	1 (2.13)		15 (3.17)		0.156 (1)		.07	
	Neurosyphilis	0 (0)		37 (7.82)		3.958 (1)		.005	

## Discussion

### Principal Findings

All people living with HIV/AIDS investigated in this study were from central China, where the main route of HIV acquisition was paid plasma donation and contaminated blood transfusion in the 1990s [19]. Later, unprotected sexual contact became the route of HIV acquisition among new infections. Moreover, unlike European and American countries, chronic HBV infection among adults in China was mainly acquired during the preschool period. Therefore, the prevalence of HIV and HBV coinfection in Hubei province shows obvious regional characteristics. This large-scale epidemiological survey used HBV markers (anti-HCV combined with HCV RNA and *T pallidum* titer level) to investigate the prevalence of coinfections with HBV, HCV, or syphilis, as well as the different intensities of propagation in individuals with different risk behaviors but the same HIV transmission route.

The recognized transmission routes of HCV are blood transmission, sexual transmission, and MTCT. The 3 main routes of HCV infection demonstrated varying levels of transmission intensity, with blood transmission being the most intense, followed by MTCT and sexual transmission. In order to determine the characteristics of HCV transmission from a blood origin, the populations in this study were further divided into former paid blood donors, contaminated blood recipients, and intravenous drug users. We confirmed that HCV infection was significantly associated with the specific exposure mode of blood contact. The chronic HCV infection rate was found to be highest in former paid blood donors (80.27%), followed by intravenous drug users (73.29%), and contaminated blood recipients (57.14%). The chronic HCV infection rate in all these groups was greater than the prevalence of posttransfusion HCV infection among individuals with β-thalassemia [21] and blood donors in sub-Saharan Africa [22]. The difference was related to the outbreak of HIV and HCV infection in the special historical period of this study. The strong relationship between the frequency of HCV exposure or the amount of blood contact and the risk of HCV infection among former paid blood donors, contaminated blood recipients, and intravenous drug users was validated using population characteristics from a specific historical time. Although this viewpoint is conceptually evident and has long been acknowledged, obtaining specific and objective data is challenging. This study, however, provides evidence on exactly this problem.

Multiple sexual partners, mucosally traumatic intercourse, and bleeding during anal contact have been found to be common among men who have sex with men, which can raise the risk of HCV infection [23]. However, it was found that the HCV infection rate in men who have sex with men was lower than in people who acquired HIV through heterosexual contact (1.24% vs 8.07%). In this study, the male-to-male contact behavior that caused the spread of AIDS in this region was relatively late, and HCV coinfection in this community was relatively infrequent, so it did not result in the broad spread of HCV in men who have sex with men.

The prevalence of HBV infections is affected by multiple factors, including epidemic HBV in the birthplace, rates of HBV vaccination, and risk behaviors for HBV exposure. In the general population, chronic HBV infection was reported as 7.18% in China [24]. A previous study showed that people living with HIV/AIDS were 7 times more likely to have been infected with HBV [25]. However, the overall rate of chronic HBV infection in this study was 10.92%, which was much lower than the above inferred values. As is well known, China is a country with a moderate prevalence of HBV, and the majority of chronic HBV infections occur through vertical MTCT during the preschool period, which is different from European and American countries. Almost 95% of people infected with HBV can completely clear the infection when it is acquired during adulthood, and only 5% to 10% of people infected with HBV will develop a persistent infection. The regional characteristics of HBV prevalence may well account for the result that chronic HBV coinfection was not as high as HCV in this study. Interestingly, we found that men who have sex with men had a higher chronic HBV infection rate but a lower HBV exposure rate compared to people living with HIV/AIDS among the heterosexual population, implying that sexual behavior among men who have sex with men was closely related to the increase in the chronic HBV infection rate, providing another reason to strengthen disease safety education among men who have sex with men. Previous research found that men are 1.5 times more vulnerable to HBV than women, implying that gender is a factor influencing persistent HBV infection after HBV exposure [26]. In addition, another key aspect that may account for the greater HBV infection rate in men who have sex with men is exposure to HBV-contaminated blood during mucosally traumatic sex. Combining all these data shows that in places where vertical HBV transmission is a key epidemic component, both the intensity of transmission and the effect of HBV exposure on the prevalence of chronic HBV infection are substantially lower than for HCV.

In this study, among people who acquired HIV by either the blood route or by sexual contact, the combinations of HBsAg/anti-HBe and HBeAg/anti-HBs were detected, which could account for the transient coexisting phases of antigens and antibodies, the overlapping infection of different subtypes of HBV, or the deletion of genes in the S region or pre-S region of HBV. An interesting finding of this study was that an unusual pattern for HBV infection (the combination of only HBsAg/HBeAg antigens) was found only among people who acquired HIV by sexual contact, which might stimulate interest in further exploration of this phenomenon. In a previous study focused on HBV vaccination among people living with HIV/AIDS, it found that anti-HBe titers in a low CD4+ group were much lower than a high CD4+ group (low CD4+ group: 462 mIU/ml vs high CD4+ group: 8834 mIU/ml), which shows that an immunosuppressed state can affect the ability to produce protective anti-HBe [27]. Although immune deficiency is a possible cause, it still could not fully explain this unusual pattern. Further follow-up monitoring in these patients will be considered to explicitly reveal whether HBV-related antibodies appear after implementation of ART. This large-scale epidemiological study based on the comprehensive HBV marker test could provide a clue for further research into the mechanism of this unusual combination of HBV markers.

As a transfusion-transmissible infection, the prevalence of syphilis coinfection among people who acquired HIV from the blood route was 8.87% in this study, which is higher than in reports on the HIV-negative populations in Nigeria (4.2%) [28], Burkina Faso (3.96%) [29], Kenya (4.3%) [30], Pakistan (0.43%) [31], and India (0.43%) [32]. Nonuse of protection in sexual relations has already been identified as a risk factor. Except for differences in sociocultural practices, geographical location, and condom coverage, people living with HIV/AIDS may be more susceptible to *T pallidum* than HIV-negative people, which is a possible reason for the observed discrepancy in the magnitude of syphilis in the different studies. High *T pallidum* and neurosyphilis titers were found to be more common in people living with HIV acquired through sexual contact, implying that safe sex education is essential to reduce the risk of late syphilis; providing easily obtained information, education, and communication materials with pictures in HIV and sexually transmitted disease clinics, even in public places, is an important measure.

Overall, there were some highlights in this study. First, the transmission mode of AIDS in Hubei province has an obvious time node, which makes the regional characteristics of this study more prominent. Second, this study used syphilis antibody titers combined with related clinical symptoms and a complete marker test of HBV, in addition to HBsAg, to conduct a large-scale epidemiological investigation on the prevalence of syphilis and HBV infection, making the observational indicators of this study more comprehensive and superior. Third, another highlight is the integration into the study of subject grouping. For example, the people with blood-borne HIV transmission were further subdivided into intravenous drug users, contaminated blood recipients, and former paid blood donors. Similarly, the people with sex-borne HIV transmission were further subdivided into men who have sex with men and heterosexual people. These divisions deepened the understanding of the intensities of propagation in people with varying risk behaviors but the same HIV-borne transmission route.

However, we recognize that our study has some limitations. Given the cross-sectional nature of the study, we are unable to observe changes in HBV markers in those patients who had unusual patterns for HBV (the combination of only HBsAg/HBeAg antigens), but further follow-up observation will be conducted in the future. Also, due to the large sample sizes involved in epidemiological analyses, HCV RNA detection was only performed in patients positive for anti-HCV, which may miss some patients with detectable HCV RNA who are negative for anti-HCV. In addition, HBV DNA screening was only done for HBsAg-positive patients, not for all people living with HIV/AIDS, which could lead to underestimating the HBV infection rate due to the presence of occult HBV infections. Finally, the level of stratified heterogeneity is something that deserves attention and needs improvement. However, given the low frequency of false negative anti-HCV and occult HBV infections in this investigation and the large sample size, the aforesaid limitations should not have influenced the overall trend of the results.

### Conclusion

HCV, HBV, and syphilis all have similar transmission routes; however, the varied routes do not carry the same weight in terms of disease prevalence. The risk intensity of HCV transmission by blood is substantially higher than that by sexual contact, and it is closely related to exposure frequency. In Hubei province, where vertical transmission of HBV is the primary route, the influence of adult HIV infection and high-risk behavior on HBV transmission is much lower than on HCV, and the range of chronic HBV infection prevalence in different high-risk groups is relatively limited. In people with sex-borne HIV transmission, high *T pallidum* and neurosyphilis titers are common. The data presented in this study provide a comprehensive picture of the prevalence of HBV, HCV, and syphilis in various populations with HIV risk behaviors, deepening our understanding that repeated exposure to unclean blood or sexual contact increases the risk of HCV and emphasizing the close relationship between neurosyphilis and repeated exposure.

As the distribution of HIV high-risk groups varies by country, so does the burden of HBV, HCV, and syphilis coinfection, and these precise comparative and analytical data serve as the foundation for future local policy creation. It is necessary to invest in preventive and control funds based on the regional distribution of people living with HIV/AIDS in order to optimize the returns.

## Abbreviations

Anti-HBc: hepatitis B core antibody
Anti-HBe: hepatitis B e antibody
Anti-HBs: hepatitis B surface antibody
ART: antiretroviral therapy
CSF: cerebrospinal fluid
EIA: enzyme immunoassay
ELISA: enzyme-linked immunosorbent assay
HBeAg: hepatitis B e antigen
HBsAg: hepatitis B surface antigen
HBV: hepatitis B virus
HCV: hepatitis C virus
MTCT: mother-to-child transmission
RPR: rapid plasma reagin circle card test
WHO: World Health Organization

## Data Availability

All data generated or used during this study are included in this published article.
